# Whole-body DXA evaluation in HIV patients

**DOI:** 10.1186/1471-2334-14-S7-O4

**Published:** 2014-10-15

**Authors:** Mihai Lazăr, Daniela Munteanu, Raluca Mihăilescu, Cătălin Tilişcan, Tudorița Mărgineanu, Ana-Maria Tudor, Loredana Benea, Anca Streinu-Cercel, Ștefan Sorin Aramă, Adrian Streinu-Cercel, Victoria Aramă, Daniela Adriana Ion

**Affiliations:** 1 National Institute for Infectious Diseases "Prof. Dr. Matei Balş", Bucharest, Romania; 2 Carol Davila University of Medicine and Pharmacy, Bucharest, Romania

## Background

After the introduction of highly active antiretroviral therapy for the treatment of HIV infection, metabolic abnormalities were increasingly observed, associated with both protease inhibitors and nucleoside reverse transcriptase inhibitors, characterized by abnormal fat distribution in the body (lipoatrophy, lipohypertrophy or both), hyperglycemia and lean tissue mass wasting. However such metabolic changes are not always treatment related, having a multifactorial etiology. Dual-energy X-ray absorptiometry (DXA) evaluates the quantity of body fat and lean tissue and their distribution, allowing the diagnosis of both quantitative and qualitative changes.

## Methods

We evaluated 58 patients (26 females and 33 males), aged between 19 and 65 years, by whole body DXA scan. Biological tests on the patients included: leptin, adiponectin, resistin, TNFa, IL6, LT CD4+, viral load. Body mass index, waist/hip ratio and waist/height ratio were also calculated.

## Results

We obtained significant correlations between the DXA data and the biological, clinical parameters, type and duration of the treatment.

## Conclusion

DXA represent an essential tool in evaluation of HIV lipodystrophy and wasting syndrome and can be used for both treatment monitoring and disease metabolic evaluation of HIV patients.

